# Views and experiences of opioid access amongst palliative care providers and public representatives in a low-resource setting: A qualitative interview study

**DOI:** 10.1371/journal.pgph.0002401

**Published:** 2023-09-21

**Authors:** Joseph Clark, Naveen Salins, Sunitha Daniel, David C. Currow, Lesley Jones, Mark Pearson, Robin Bunton, Joseph Mankel, Christopher Braithwaite, Marianne M. Gilchrist, Miriam J. Johnson

**Affiliations:** 1 Wolfson Palliative Care Research Centre, Allam Medical Building, University of Hull, Hull, United Kingdom; 2 Department of Palliative Medicine and Supportive Care, Kasturba Medical College Manipal, Manipal Academy of Higher Education, Manipal, India; 3 York and Scarborough Teaching Hospitals NHS Foundation Trust, York, United Kingdom; 4 Faculty of Science, Medicine and Health, University of Wollongong, Wollongong, Australia; 5 Hull York Medical School, Allam Medical Building, University of Hull, Hull, United Kingdom; University of the Philippines Diliman, PHILIPPINES

## Abstract

Opioids (e.g. morphine) are affordable, effective interventions for cancer-related pain. However, equity of access to this key medication remains a global challenge, particularly in low- and middle-income countries. We aimed to explore views of palliative care providers and public-representatives about opioid analgesia access in two States in India. We conducted a qualitative study using semi-structured interviews. Transcribed audio-recordings were subjected to thematic analysis using a Framework Approach. Palliative care providers and public-representatives were purposively sampled from services reporting consistent opioid availability and prescribing (≥4kg per annum) from Karnataka and Kerala. Twenty participants (doctors (10), nurses (4), pharmacists (2), service managers (2) and public-representatives (2) were interviewed. Three themes were identified: 1) Attitudes and awareness: opioid treatments are perceived as end-of-life (last days/weeks) interventions; fears of addiction and misunderstanding of pain management goals limit access. 2) Expected and unexpected inequities: patients/carers from lower socioeconomic strata accept doctor recommendations if opioids are affordable, more educated patients/families have reservations about opioids, delay access and perceive expensive medicines as better. Non-palliative care specialist doctors have negative entrenched views and require specialist training. 3) Experiential learning–positive experiences can positively alter attitudes (e.g., participants in Kerala report improved attitudes, awareness and understanding influenced by exposure and community awareness, but experience can also reinforce perceptions as end-of-life care. Entrenched negative views are reinforced by poor experiences while positive experiences improve attitudes. To promote access, opioid prescribing must be needs-based rather than prognosis-based. Addressing the lack of training for non-palliative care workforce would help overcome a major barrier.

## Introduction

Access to opioids is a human rights issue and key global health inequity [1]. Opioids such as morphine are listed as Essential Medicines by the World Health Organization (WHO) and are effective for managing moderate-severe pain, chronic refractory breathlessness [2] and trauma, and improve quality of life [3]. Without appropriate treatment, people with moderate-severe pain due to cancer and other life-limiting illness experience severe suffering, with associated reduced function and quality of life [4].

Activists and global health agencies have prioritised increasing access to opioids for moderate-severe cancer pain [5]. As part of efforts to achieve Universal Health Coverage countries, World Health Organisation member states aim to ensure that access to pain treatment is needs-based and not dependent on stage of illness [6]. However, despite the low cost of opioids, most people worldwide who would benefit do not receive them [7].

A complex interaction of socio-regulatory factors influences opioid availability in low resource settings [8]. Accessibility, however, is associated with physicians’ willingness to assess pain and prescribe opioids, and patients’ willingness to adhere to prescribed treatments. In Asia, concerns about regulatory redress limit physicians’ willingness to prescribe opioids, despite knowing their benefit [9]. Negative public discourses drawn from opioid over-consumption in some high-income countries, associated with addiction and illicit drug use may also influence patients’ and families’ compliance with prescriptions [10].

Reports from India, which has one of the most established cancer services in South Asia, show few people with severe pain due to cancer gets the necessary analgesia [11]. A four-centre study in northern India showed that, despite 88% of people with cancer reporting pain, only one-third received adequate pain management [12]. In the context of serious inequities in access to pain treatment in India, there are pockets of better opioid availability in individual clinical teams in some States (e.g., Karnataka, Kerala) [13]. Working under different governance, some services have navigated difficulties and developed solutions to improve availability and accessibility of opioids, although challenges may remain. Experiences of palliative care providers and public representatives from clinical teams who provide a good level of opioid availability and accessibility in the context of national inequities, would usefully inform other States in India and low-resource countries planning to overcome obstacles to implementation of pain management. In this initial study, we aimed to:

- explore the views, attitudes and experiences of palliative care providers and public-involvement representatives towards opioids in two States in South India;
- identify implementation practices which maximised equity of access to opioid analgesia for cancer-related pain.

## Materials and methods

### Study design and setting

Our qualitative interview study used semi-structured interviews to explore perceptions and experiences of opioid access amongst a representative sample of professionals involved in facilitating opioid availability and accessibility and public representatives in two States in India. Our research was undertaken in context of implementation of the 2014 Amendment to Narcotic Drugs and Psychotropic Substances Act, which details specific responsibilities relating to: procurement, storage, prescribing and dispensing of opioids [14]. We report our study following the Consolidated Criteria for Reporting Qualitative Research (COREQ) guidelines [S1 Checklist] [15].

**Box 1. Setting–focus on Karnataka and Kerala**

*Study setting—focus on Karnataka and Kerala*

Our sample was drawn from palliative care teams in Karnataka and Kerala with sufficient experience of opioid prescribing. To define this, we used a proxy measure of an annual prescription rate of morphine per service of 4kg or more. This amount was agreed by clinicians in India within the research team as a quantity indicative of a service routinely prescribing to meet the pain-control needs of cancer patients.

Karnataka has a population of approximately 70 million and enacted a State policy for palliative care in 2016 [16]. Palliative care and opioid treatments are commonly provided in a hospital setting, with 16 known palliative care centres that includes hospital and hospice settings across the State [17]. Commonly, prescriptions are made in an inpatient or outpatient setting and prescriptions are dispensed in hospital pharmacies. Kerala has a population of 35 million and was the first State in India to have a specific policy for the implementation of palliative care (2008) [18]. More than 400 non-governmental organizations support the delivery of palliative care in Kerala, which is recognised as a global leader in providing community-based palliative care and opioid provision [19]. Palliative care in Kerala is delivered in a number of settings including government hospitals, private hospitals, and community-based NGOs and there are more than 400 organisations that support the delivery of palliative care [20]. Opioids are widely available in the State and are commonly prescribed in an inpatient or outpatient hospital and hospice settings by approved providers affiliated with a Recognized Medical Institution(RMI)

### Identification, recruitment and consent

Potential participants were identified from teams with sufficient experience of opioid prescribing for people with moderate-severe cancer pain. Eligible public representatives were lay people with experience of providing informal care, either in support of professional services as volunteers, or as family members.

We identified individuals through existing contacts and networks (e.g. Indian Association of Palliative Care, Pallium India). Potential participants were invited and received information about the research by email (SD, NS). Those eligible were invited to discuss the study in more detail with our investigators in India, who contacted them to provide additional information and answer questions about the project and/or project team. Individuals willing to take part were asked to provide informed recorded oral consent prior an audio-recorded interview.

### Sampling approach

We aimed to recruit a sample of 16–20 using a purposive sampling framework including perspectives of doctors, nurses, pharmacists, service managers and public representatives [21]. We prioritised achieving a balance of perspectives based upon: years of experience, sex, profession and State. Due to difficulty identifying a public representative for one of the States, we recruited a public representative with experience of opioids from the perspective of being an informal carer for a relative, from a third State (Maharashtra).

### Data collection and management

Data collection took place in context of a wider programme of work exploring implementation of narcotics regulations in Karnataka and Kerala. An experienced qualitative researcher (woman) based in the UK (LJ, PhD) conducted semi-structured interviews in English using a pre-defined and piloted topic guide [S1 Text]. Our interview guide was developed drawing upon the published literature, the research team’s expertise, and was informed by the Consolidated Framework for Implementation Research, adapted for use in Low and Middle-Income Countries [22].

Interviews were conducted between 10.08.2021 and 23.12.2021 and lasted between 30 minutes and one hour. Interview data were recorded and stored compliant with General Data Protection Regulation 2018. Interview recordings were transcribed verbatim by two researchers and checked for accuracy by a research administrator (MMG). Interviewee responses were anonymised and participants allocated a unique study identifier, noted alongside their role and their State.

### Data analysis

Thematic analysis was conducted using a Framework Approach [23]. Initial coding of three transcripts was undertaken by three researchers to develop a preliminary analytical framework informed by the Consolidated Framework for Implementation Research, adapted for use in Low and Middle-Income Countries (JC, RB, SD) [23]. The analytical framework was then refined by our interdisciplinary team and applied to all subsequent transcripts by JC, adding any additional codes necessary to reflect participant viewpoints. Analytical themes were then developed through team discussion. Themes are reported, drawing out key similarities and differences between responses from Karnataka and Kerala. Data were analysed using NVIVO 12 Pro [24].

### Ethics

We applied recommendations on good ethical conduct of the Economic and Social Research Council (ESRC) Guidelines for Research [25]. Our study received ethical approval from Hull York Medical School ethics committee [REF 21 11, 18/02/2021] (United Kingdom), and the Kasturba Hospital Institutional Ethics Committee, (REF 224/221, 30/03/2021). The study was registered with the Clinical Trials Registry of India (CTRI/2021/04/033156, 19/04/2021). As it was an international study, the study required an additional approval from the Indian Council of Medical Research (ICMR) and Health Ministry Screening Committee (HMSC) in India (REF 2021–0126, 04/08/2021).

## Results

Twenty interviews were conducted, with a good balance between: experience, sex, profession and State of occupation [Table 1].

**Table 1:** Participant characteristics.


Number of years involved in palliative care	• <5 years • 5 to 10 years • >10 years	5 5 10
Sex	• Men • Women	7 13
Profession	• Doctor • Nurse • Pharmacist • Manager • Public representative	10 4 2 2 2
State	• Karnataka • Kerala • Maharashtra	8 11 1


Three themes were developed, each with relevance to patients, family members and healthcare professionals but varying impacts for different groups [Table 2]. We present themes with illustrative quotes where appropriate.

**Table 2:** Themes and sub-themes.

**Attitudes to opioids and palliative care**	Misperceptions of purpose of treatment	Patients, family members and healthcare professionals
	Fears of addiction and dependency	
	Side-effects and sedation	
**Expected and unexpected inequities**	(Mis)education	
	Cost of medicines	
	Hierarchy of medicine	
**Experiential learning**	Challenging and entrenchment of views	

### Attitudes to opioids and palliative care

Negative attitudes towards opioids persist amongst patients, medical professionals and policymakers, e.g. risks of addiction, concerns over respiratory depression and amongst patients that opioid treatments were only for people at end-of-life.

### Patients and families

Participants reported entrenched negative attitudes and misperceptions of palliative care amongst patients and family members in both Kerala and Karnataka. The offer of palliative care and opioid medications were both perceived by patients and family members as indicating death was near.

*Many patients*, *or their bystanders or relatives involved*, *they—some of them tend to think that now a person is put on opioid that means he is going towards end of life*. [OCP18, Volunteer, Kerala].

Accordingly, participants reported low understanding amongst patients of the purpose of opioid medications.

*Some [family members] ask*, *‘You are just going to sedate my father by giving this morphine*?*’ Yeah*. *Certain questions said that because they have heard of it before*. *Like you give this medicine*, *they take their medicine and they just sleep off because not knowing that pain again*, *get up from the sleep*, *they give the morphine again and he goes to sleep*. *Is that what they are going to do*? *So we need to counsel them about the situation*. [OCP6, doctor, Kerala].

Many participants discussed fears of addiction. In Kerala, one participant suggested that fears had reduced over time. In Karnataka, another reported that negative perceptions led to poor adherence, or avoidance, of prescribed medicines.

*Generally*, *as in many other places there is some stigma [about opioids]*, *particularly amongst the relatives and family*. *We suggest morphine and some of them feel that this is the last resort*. *Are we giving up*? *But the concern about addiction it has calmed down*, *we don’t hear a lot of questions about that*. [OCP10, Doctor, Kerala].

*Fear of opioids is there*, *ma’am*. *So*, *they are thinking that it would cause the addiction*, *it will kill them*, *so…*. *And moreover*, *you know*, *they are not even properly expressing their pain stage*. *They are experience—what kind of a pain you’re experiencing and whether they are experiencing pain or not because they’re hiding*. [OCP15, Nurse, Karnataka].

### Healthcare professionals

Negative attitudes and perceptions amongst patients and families are contextualised by misperceptions amongst non-palliative care professionals. Many participants in Karnataka and Kerala cited that non-palliative care physicians shared patients’ views that opioids are for end-of-life only, or that opioids cause dependence and/or respiratory depression.

[Non-palliative care physicians are worried about] *managing the side-effects*, *thinking that a very low dose [of morphine] could suddenly trigger like respiratory depression*, *and that they might not be able to handle it in a resource-limited setting*, *in case the patient requires intubation*, *if that event occurs*. [OCP13, Doctor, Karnataka].

Concerns about morphine were reported as influencing treatment decisions and referrals to services. Here, a mixed picture was presented. In Kerala, participants referred to these issues, but suggested attitudes were changing and referrals from other medical disciplines were increasing. There was less evidence of improved knowledge in Karnataka, where pain relief was actively avoided by non-palliative care clinicians: patients were only offered step 2 pain medications, or symptomatic patients were recommended additional disease-modifying therapies only. In addition to opioid use for pain, participants reported frustration that non-palliative care clinicians were reluctant to administer morphine for other symptoms for which there is an evidence base, such as breathlessness.

*Even when the patient was comfortable with prescription of morphine*, *there was lot of reluctance and resistance from the other professionals*. *Yeah*, *it was there*. *So*, *they used to discourage patients from having morphine*, *saying that ‘this is an end-stage medication*, *your pain is not that severe*, *so that you don’t have to do this’*. *But nowadays that is not there*. *People have even professionals have come forward with prescription of morphine*. *Even the pulmonologist*, *even the respiratory care physicians*. *[OCP3*, *Doctor*, *Kerala*].

*I can give you an example wherein if you have a patient goes to a cardiologist and his ejection fraction is about 25% and he would be breathless and panting*. *If you [a palliative care doctor] just tell him*, *‘I’ll give him just about 5 mg of oral morphine*, *he would be much better with his breathlessness and things like that’*. *Cardiologist would actually say*, *‘I don’t think so’ and would require that he would be given the second group of third line of medications for the cardiac work to improve*, *rather than actually relieve a symptom burden at all*. *[OCP12*, *Doctor*, *Karnataka*].

### Expected and unexpected inequities

Education and socioeconomic status were reported as key determinants of attitudes to pain treatment and palliative care amongst patients, families and healthcare professionals. An unusual relationship between level of education and negative attitudes to opioids was identified. Patients with low-levels of education (typically socioeconomically-deprived) and clinicians who have received specialist training in use of opioids were reported as having positive attitudes, whereas people with high levels of non-specialist education (typically more wealthy patients and non-palliative care medical professionals) held negative attitudes.

### Patients and families

Participants perceived that financially less well-off patients lacked preconceptions about opioids, commonly trust their doctor and accept opioids. Wealthier patients with preconceived ideas were more likely to have negative perceptions, and thus typically receive palliative care and pain treatment at a later stage, or are less compliant with prescribed opioids.

*Most of our patients are daily wage patients*, *below poverty line patients or average lower middle-class patients*. *They don’t have any problem in taking morphine*. *They don’t have any problem and they comply*. *They comply with us and they give exact report of what happened–what happened to them after taking morphine*. *But this educated… the so-called educated patients and have got so many worries and doubts and all…*.*so*, *they may not comply with your suggestions or prescription and some of them feel that once you take morphine*, *you’ll be sedated*, *you’ll be sleeping all the time*. *You will be sleeping all the time and you become an addict*, *also*. [OCP7, doctor, Kerala].

Unexpectedly, negative attitudes were less likely to be related to low levels of education. For patients, this was commonly because they had done their own internet research to reach conclusions.

*Literate people and people who have really done well in life*, *they will Google everything and they would know better than you*. *They come and discuss about all the adverse effects*, *sometimes you even worry about having an interview with them because they definitely know more about what they expected and then about the medications that they’re going to give them*. [OCP2, doctor, Kerala].

The finding that more affluent, more educated people are less likely to accept opioids and palliative care was problematic. In Kerala, most opioids were reported as without cost, enabling patients from all economic strata to access pain treatments. In Karnataka, participants reported access to pain treatment and palliative care mirrored broader healthcare inequities, determined by the ability to pay for medical care.

*Some are very poor*, *ma’am so patients don’t have the ability to pay for that basic analgesia*. *So*, *some are just avoiding*, *and they don’t want to take a prescription they don’t want to*, *you know*, *uh*, *they want to take adequate analgesic therapies and it’s due to the financial crisis*. [OCP15, nurse, Karnataka].

### Healthcare professionals

Non-specialist medical education was perceived by participants as propagating negative attitudes to opioids. Medical professionals with negative attitudes towards opioids use a generalist level of knowledge of pain management to avoid strong opioids. Additionally, this grounding in non-specialist palliative care education was perceived as *increasing* resistance to educational learning and attitude change.

*So*, *from the perspective of the healthcare workers people who have got some form of training with opioids have accepted much better*. *But people who have not had any training in opioids will not accept it*. *If I do convert*, *giving you a simple example*, *400 mg tramadol*, *yeah*, *is almost equal to 40 mg of morphine*, *roughly 40 to 50 mg of morphine*, *and I say I can give 10 mg*. *At least they would accept 400 mg tramadol easily compared to 20 mg morphine*, *which is even half of that 400 mg conversion of tramadol by my own colleagues*. *And if some patient comes up with some form of dizziness*, *something else the first drug they’re going to strike out is morphine*. [OCP12, Doctor, Karnataka].

*The professional is brimming with knowledge which will have to be unlearned for any new learning to come in*. *‘‘Morphine*? *Respiratory depression*. *Morphine*? *Addiction*.*” It’s like a brick wall*. [OCP1, doctor, Kerala].

Many participants highlighted the need to educate non-palliative care physicians regarding opioid prescribing, to change attitudes and increase human resources for prescribing. However, participants reported challenges. Medicine is a hierarchical profession, in which palliative care is assigned lesser recognition, making it challenging to engage professionals from other healthcare specialties. Additionally, length of available training courses was perceived as a barrier to engaging healthcare professionals from other disciplines, reluctant to take too much time out of their usual work for training. One participant argued strongly that the basic skills to safely prescribe opioids could be taught in a very short period of time.

*I’ve been arguing we should cut down the training*, *because what happens pretty often*, *at least nowadays*, *is people are not coming forward to do the training because it is ten days*. *Particularly the oncologists that we see*, *they’re not happy about the ten days*. *He or she says that*, *‘OK*, *we have been handling more harmful and more difficult drugs*. *Maybe one*, *one and a half days would be adequate’*. [OCP10, Doctor, Kerala].

The same participant distinguished between the skills necessary to provide palliative care and those required to safely prescribe opioids, arguing that existing training regimens lengthened training courses unnecessarily by conflating the two skills.

*If they know how you break bad news to somebody*, *that’s good*. *But what is adequate*? *What is absolutely essential*? *So*, *if you cut it down to*, *if you take it away from the package of Palliative care training and say*, *‘OK*, *for the opioid licencing you need to know the opioid drugs and other drugs available how you get it*, *how to store it*, *how to use it*,*’ that can be a separate training platform*. *But that’s not happening*. [OCP10, Doctor, Kerala].

### Experiential learning and community engagement

Exposure to positive effects of appropriate opioid treatment was perceived as important in improving attitudes among both patients and healthcare professionals. However, participants acknowledged that experiences could negatively influence ‘learning’ and reinforce negative perceptions. Participants described the benefit of and need for more community engagement, noting that medical professionals’ attitudes are also shaped by their communities.

### Patients and families

Many participants reported that attitudes were changed by witnessing or experiencing benefits of opioid treatments. One participant described the transformative experience of a patient who had not received input from palliative care, and positive experience once they were identified and opioids were prescribed. The patient was in the Intensive Care Unit with severe dyspnoea and observed a patient with the same symptom experiencing benefit from a palliative care doctor and requested palliative treatment.

*…Then I prescribed morphine and let me tell you this miracle I discharged her on the morphine pump*, *with morphine and midazolam*, *and she wanted to go home*. *And we were all sure that she was going to die within a week or two and then three weeks after I get a picture*, *a series of snaps of this lady smiling in front of the biggest shopping mall we have here*. *What a shock it was to me*. *Amazing*, *I only gave her the morphine so that she would die in peace*, *but God had other plans*. *… The quality of life was superb*, *she died after three months and she was happy*, *she had a good life*. *Amazing*, *to me it was like a miracle*. [OCP2, doctor, Kerala].

However, one participant acknowledged that patient perceptions of opioids as end-of-life treatment may have been shaped, or learned negatively, through previous experiences.

*So*, *it depends upon their own previous exposure to opioids in terms of*, *could be a patient who was a caregiver before and would have seen somebody receiving morphine about 10 years or 12 years ago*, *wherein it was the patient was terminal and they have used morphine*. *He would not accept once we initiate morphine*, *saying that ‘I’m not terminal’*. [OCP12, Doctor, Karnataka].

To challenge negative perceptions, participants highlighted the importance of engaging communities and sharing positive experiences. Participants in Kerala reported successes using community-based approaches, in particular through social and other media. Improved awareness, attitudes and understanding in the extensive network of volunteers in Kerala is a particular success of community-engagement. Despite ongoing challenges in Karnataka and Kerala, participants were outward-looking, promoting awareness at national level. Challenging some patients’ fatalistic beliefs about the inevitability of suffering during illness was deemed a key challenge, along with the need to foster attitudes of receiving appropriate pain treatment as a right. To do so, there was acknowledgement that community engagement is a specialist activity.

*Public engagement is the key for improving health*. *Those are things that we have been doing amateurishly*. *Maybe more professional approach is necessary*. *We haven’t worked out yet exactly how to do it*, *but if I had the right resources and the right people*, *I would be writing commentaries and experience sharing from the public from the people lived with lived experiences*. [OCP1, doctor, Kerala].

### Healthcare professionals

Participants all highlighted the need for increased human resources for prescribing and promoted further education. However, despite some participants expressing optimism for broadened participation in training through online delivery, the importance of exposure to the benefits of opioids was highlighted as essential for challenging attitudes.

*So*, *I would say*, *a regular MO [medical officer] would be fascinated if he sees in the ER [Emergency Room] when a patient is screaming in pain and you do an IV titration [of morphine] and the patient comfortable within about 10–15 minutes*. *So that leaves a very long impact on your learning experience*. [OCP12, Doctor, Karnataka].

Finally, while many participants emphasised attitudes could be changed through experience, others emphasised how deeply ingrained and difficulty to alter attitudes were,–even amongst medical professionals. One participant reported that a colleague within their hospice, currently experiencing severe pain from cancer, was hesitant to accept an opioid prescription, despite professional exposure:

*Unfortunately*, *my nurse*, *she was the one who was holding one key to the morphine all these years*. *She has cancer breast and she’s under treatment*. *And she was a terrible pain*, *but she just refused to take morphine and she’s been dispensing morphine*, *how many years now*? *Somehow*, *she’s got this phobia that once you take morphine*, *you’re all going to die… She knows about morphine*, *she even takes a class for the auxiliary nurses*, *a person like that refuse to have morphine even when the pain was terrible*. *She said*, *‘No*, *I can’t*. *I think it can be controlled with Tramadol and paracetamol’*. [OCP2, doctor, Kerala].

## Discussion

Our data show how prescribers in settings where opioids are increasingly available are attempting to implement opioid treatments before patients are in the last few weeks/days of life, but face persisting negative attitudes to opioid analgesia amongst patients, families and healthcare professionals. Patients and families commonly associate opioids with end-of-life care and risk of addiction. Perceptions are influenced by lack of knowledge, but also previous experiences. Previous experiences may be positive, witnessing benefits of opioids, and negative, where late prescribing reinforces associations with end-of life. An unusual inequity is present. Less-educated patients access opioids earlier (if cost/pricing permits) due to fewer preconceptions and greater trust in healthcare professionals. More educated patients and families understand the minor risks of prescribed opioids, but have little understanding of the benefits: they delay or resist accessing them. It is reported how people with fewer concerns or less knowledge about medications are more likely to adhere to medical advice regarding prescriptions [26]. However, our novel finding that more educated people are more likely to have negative perceptions of *opioids* should be explored in other low-resource countries aiming to increase accessibility of opioids so that patient-level concerns regarding uptake of opioids can be anticipated and addressed.

Non-palliative care healthcare workers avoid opioids despite an increasing understanding of their benefits. Skills in non-opioid pain management with serious concerns about perceived clinical risks of prescribed opioids lead to neglect of symptom management or use of weak opioids only. Participants highlighted the need for specialist training to counter negative attitudes, although opioid prescribers working alongside non-palliative care physicians also shifts attitudes, and increases symptom management referrals.

Our findings bring into sharp focus the importance of attitudes in influencing opioid accessibility. The Lancet Commission on the Value of Death highlights how unequal deaths follow unequal lives [27]. This is evident, when costs of opioid or of travelling to prescribing centres limit access to treatment [28, 29]. However, we identify an additional challenge to improving opioid access in low-resource settings, where some awareness of opioids may be a greater barrier than no awareness at all.

Healthcare professionals’ attitudes are shaped within communities as well as through medical education [30]. We highlight that previous experiences strongly influence attitudes to opioids; patients are likely to perceive opioids to use only in their last days or hours if this has been their previous experience with other family members. By contrast, experiencing or witnessing the transformative benefits of opioids for patients with moderate-severe pain influences perceptions positively–for patients, family members *and* healthcare professionals [1]. Our finding that knowledge and attitudes had improved in Kerala over time suggests that the establishment of new services may not immediately improve access to analgesia, but that community exposure may influence knowledge and attitudes over time.

Our findings have wide implications for accessibility of opioids in countries aiming to improve availability and accessibility of opioids. Reports that a moderate level of health literacy regarding opioids commonly means associating opioids with addiction and distressing side-effects highlights the pervasiveness of negative public discourse. Academics and regulators must exercise caution when highlighting risks associated with opioids and include statements regarding unmet need and benefits of opioids when discussing the complex issue of balancing availability and restriction [31]. This is particularly important for high-income countries, where framing of opioids in terms of the ‘crisis’ relating to opioid-addiction and dependence has the unintended consequence of contributing to a public discourse which reduces appropriate access to opioid treatments of people living in extraordinarily difficult circumstances in low resource settings [32]. Two issues arise for prescribers encountering patients with embedded perceptions of opioids as end-of-life treatments only. One, practice must promote experiences which include needs-based opioid treatments prior to the dying phase and consider for symptoms other than pain (e.g. chronic refractory breathlessness) [2] to influence attitudes more broadly [33]. Second, there must be open communication regarding goals of opioid treatments. Opioids *are* appropriate medications for moderate-severe pain–and other symptoms (e.g. breathlessness)–during progressing illness and at the end-of-life. When prescribing opioids at the end-of-life, clinicians must clearly communicate that opioids may help make their experience more comfortable–but will not shorten life if titrated appropriately.

Finally, our finding that non-specialist medical education may raise barriers to opioid prescribing indicates the necessity of integrating education regarding opioid prescribing within all relevant clinical specialties. A short course providing proportionate, ‘need to know,’ specialist opioid education should be mandatory in educational curricula. Including palliative care within medical undergraduate curricula in India is a significant step forwards, but this requires continued monitoring to ensure implementation and evaluation to promote positive attitudes to opioids for symptom control [34].

### Strengths and weaknesses

A key strength of our study is its inclusion of views from a broad range of palliative care providers and public representatives in two States in India with differing levels of opioid accessibility. This lets us identify issues experienced in context of different levels of service development and highlights opportunities to improve attitudes. Conversely, a weakness of our study is associated with this, in that issues in these two States are likely dwarfed in other States and low resource-settings with little, or no, availability and accessibility of opioids at all. A further limitation is that due to challenges present due recruitment taking place during the COVID-19 pandemic, we were unable to recruit additional public representatives.

## Conclusion

Attitudes to opioids change over time, but experiences and exposures can positively or negatively shape attitudes. In the presence of challenges, palliative care practitioners must ensure that the service they provide promotes needs-based access to opioid treatments to positively shape attitudes over time. Appropriate awareness of the utility and benefit of opioids, with appropriate use, will reduce unnecessary suffering.

## Supporting information

S1 Checklist Consolidated Criteria for Reporting Qualitative Research (COREQ) checklist. (DOCX)

S1 Text Topic guide. (DOCX)
