# Supplementary material for: Views and experiences of opioid access amongst palliative care providers and public representatives in a low-resource setting: A qualitative interview study
Source: PLOS Glob Public Health. 2023 Sep 21;3(9):e0002401. doi: 10.1371/journal.pgph.0002401 (PMC10513320; doi:10.1371/journal.pgph.0002401)
Supplement: S1 Text — (DOCX) [file pgph.0002401.s002.docx]

**Interview topic guide on how to ensure opioid availability for people with moderate to severe cancer pain**

**Preamble**

Can I start by asking what you know about what the research is about and if you have read the Information Sheet?

*Further explanation added if need be.*

Yes we are interested in your experience of access to opioids for people with moderate-severe cancer pain.

Researcher: reads all aspects of the participant oral consent form and asks participant to respond verbally to each item [insert version] for capture by digital recording.

**Topics to be covered during interview, with a focus on how barriers have been overcome and remaining challenges.**

1. **Background and experience.**

Job role. Experiences of working in palliative care.

1. **Opioid procurement**

Types of opioid available in service. Stocks and supply. Processes necessary to ensure continuity of supply.

1. **Storage of medicines**

Storage, security and monitoring.

1. **Prescribing**

Symptom assessment. Dosage and duration. Safety monitoring.

1. **Training and support**

Training opportunities and support available.

1. **Dispensing**

Relevant legislation and regulations.

1. **Patients’ access to prescribing**

Patient/carer attitudes to opioids. Information needs and practical challenges.

**Final thoughts**

- Is there anyone at your service or elsewhere you think we should speak to?
- Is there anything else you can think of that would be useful for us to know?
